# Six Tips on How to Bring Epic Wins to Health Care

**DOI:** 10.3389/fpsyt.2017.00264

**Published:** 2017-11-30

**Authors:** Anna Sort, Yasser Khazaal

**Affiliations:** ^1^University of Barcelona, Barcelona, Spain; ^2^PlayBenefit, Barcelona, Spain; ^3^Research Centre of the Montreal University Institute of Mental Health, Montreal, QC, Canada; ^4^University of Geneva, Geneva, Switzerland; ^5^Geneva University Hospitals, Geneva, Switzerland

**Keywords:** games for health, gamification, Internet treatment, mental disorders, Internet

## Introduction

Gamification and game-based treatment for mental health disorders are growing topics in the scientific literature. In health care, gamification is the use of various game processes in non-game contexts to engage users in behaviors that aim to improve health-related outcomes (1). In other words, gamification seeks to create motivational experiences that engage users in gameful actions and fulfill the human need for fun.

Several benefits are expected from the development of games for mental health, such as an increase in the reach of mental health interventions (appealing effect) and a reduction in the attrition rates of Internet-based interventions (engaging effect) (2, 3). Furthermore, games are expected to help behavioral change *via* situated learning (4)—which derives from achievements in participatory knowledge in immersive contexts—possibly involving activity, training, and social practice (2, 3, 5–7). Over the past 5 years, a number of studies on board games and digital games have suggested that approaches such as the gamification of therapeutic processes or casual games used for therapeutic purposes have the potential to promote cognitive and behavioral change and improvements in symptoms (8–21). A well-designed recent meta-analysis (22) that included nine randomized controlled trials (i.e., 674 participants) assessed the effectiveness of digital serious games (SGs) for the symptoms of mental disorder. The authors found a moderate effect on the improvement of symptoms that favored SGs over controls. They concluded that SGs may be effective in reducing the symptoms of mental disorder and asked for additional studies in this promising field.

The assessed games varied widely in their aims (e.g., psychoeducation, cognition training, goal-oriented, problem solving), format (e.g., involving avatars, fantasy world, personalization, exergames) and mechanisms. Some of these games were designed for treatment, while others were casual games (e.g., “Tetris”) used in expectation of a therapeutic effect (22). Unsurprisingly, therapeutically designed games vary widely in their theoretical background depending on their specific aims. For example, the game called “Let’s Face It!” is based on the theory of enhancement of recognition skills and aims to increase facial recognition abilities (23), the game called “Sparx” is based on cognitive behavioral therapy (CBT) and seeks to treat depressive symptoms (10), and “Michael’s Game,” which is based on the principles of CBT for psychotic disorders, aims to reduce delusional conviction (8).

Some reviews have reported, however, that most health-related games lack several typical immersive gamification features (24) and have a limited theoretical foundation (25). In addition, despite promising results and the *a priori* engaging nature of games, high rates of attrition and a decrease in game use over time have been previously reported (26, 27). For instance, none of the 1,622 adolescents participating in a study on a computerized game designed for reducing binge drinking completed the five sessions of the game (28). Thus, although game design provides flexible opportunities to promote behavior change, target goals need to be reached in order for games to be successfully adopted by users.

Despite the attractiveness (29) and potential of games, the processes underlying game design have not been reported in detail in most game-related studies. This weakness has commonly been reported in the literature for behavior change interventions (30) and has led to a lack of guidance on how to practically design such interventions, particularly in health-related games (31).

The present opinion paper aims to outline possible steps to follow in order to implement gamification in health care by using adequate solutions. SGs must demonstrate the transfer of learning (to be “serious”) while, at the same time, remaining engaging and entertaining (to be “games”). The balance between fun and educational measures should be targeted throughout game development, starting from the design phase. Yet, despite the potential of digital games in terms of interactivity, immersion, and engagement, further studies are needed to understand how to better design, administer, and assess such games across different learning contexts and targets (31, 32). To date, the insufficient integration of behavior change and game design principles is one of the biggest issues with SGs (27).

The proposed tips for gamification are as follows.

## Know What Your Goals are

Translating goals into game design mechanisms and processes is the pinnacle of gamification (31). In other words, one of the most important steps to ensure a successful outcome of the engagement design is to know what your goals are (i.e., the game goals and the real-life outcome goals). Accordingly, users’ engagement is expected to increase when their expectations match the goal of the intervention (5). To move from general, non-specific goals to more specific goals and to refine goal setting (33), it might be useful to involve designers, clinicians, and end users in collaborative workshops.

## Put the User First

As obvious as it may sound, this second tip is too frequently disregarded. Giving priority to the client’s or the patient’s experience is a necessary condition for meeting end users’ needs effectively (3). Designers often do a great job in thinking about how to get the best results, but then completely forget to consider how to interest older adults in using a tablet and a medical device, how to teach them to use the device, and how to warn them that they even have to use a device. Therefore, the project might be doomed to failure before it even begins. By putting the users’ experience first, you will understand exactly what you want the users to go through, as well as what they are going through. As a result, you will ensure that game processes are designed to suit the users’ needs and abilities.

This is a crucial step in developing, planning, and performing actions that succeed in promoting goal attainment in the game (33). It involves considering the main determinants of the given behavior, possible short- and long-term concurrent goals, practical obstacles, and the correspondence between the goal and the users’ self-image, self-compassion, and values (34–37). When you meet with users, adopting a detailed approach may help you precisely describe the steps they take and their level of stress with each step. It is important to ask them to describe the steps that they expect next for their benefit, as well as any “crazy ideas” they may have. In the end, you might gain valuable insights into what users are doing, what they would like to happen and what they would love to happen.

Furthermore, game designs must be tailored to the needs and capabilities of the target group in order to enhance motivation and fun (38), self-efficacy (39), and adherence (38). For instance, recently published design recommendations for older adults’ SGs have taken age-related cognitive change into account (40). Giving priority to the user’s experience could be achieved by using participatory, user-centered design, i.e., continuously involving users at every stage of development or formative research steps and collecting information from the target group to ensure that game processes are in tune with their needs and capabilities (41). Despite its theoretical importance, the impact of participatory design (PD) on game engagement and effectiveness is nevertheless inconclusive and is likely to depend on the type of PD and focus. Nonetheless, involving users in the development of key game components may be more effective than involving them in esthetic appreciation (41, 42).

## Use Behavior Change Theories to Inspire Your Gamification

When thinking about “gamifying” something, bear in mind what behavior you want to change. Do you want people to be using something they are not using now? Do you want them to increase the number of times a day they use something—something they currently use only once a day? Do you want to introduce something new into their routine? Are the people you want to help facing specific competing impulses or inhibitions related to the game’s main pursued goal? Are knowledge, specific beliefs, expectations, or barriers influencing the given behavior? Is the game addressing motivation, self-regulatory capacity, or any other specific skill? These are concerns that must be addressed to define what should be achieved in terms of behavior and to reach your goal. To do so adequately, using a given behavior change theory (30, 43) provides helpful guidance in the game design process (44).

Considering the variety of behavior change theories and their differences in focus and construct—such as the Health Belief Model, the Theory of Planned Behavior, and self-control and willpower-related models (43, 45, 46)—it is important to choose the most appropriate guidance in line with the game’s goals.

Some studies found that explicit reliance on behavior change theory and its related techniques (47, 48) was associated with better outcomes (49, 50). Unfortunately, most of the available health-related digital interventions did not explicitly rely on such theory (51–55). Moreover, specific behavior change techniques may vary across goals and topics (47, 48, 56, 57). However, some behavior change theories such as the Self-determination Theory (58), Fogg’s Behavior Model (59), and Social Cognitive Theory (SCT) (60) may provide guidance about game development across health-care sectors.

Self-determination Theory (58) highlights the importance of competence (the need to control the outcome, to experience mastery), relatedness (being connected with others), and autonomy in the change process, as well as of a coherent framework in game design (5). Fogg’s Behavior Model states that behavior change is a function of three fundamental elements: motivation, ability, and trigger (e.g., a motivating element, a facilitator, a signal) (59). SCT (60) focuses on how to change self-efficacy—a main determinant of behavior change—when facing barriers in achieving goals and suggests different solutions such as vicarious experiences. A smoking cessation game, leading to an increase in self-efficacy based on game processes and learning situations related to the barriers usually faced by people trying to change their smoking behavior, provides a valuable illustration of that process (11).

To improve the articulation between the different components of game design for behavior change, Starks proposed a cognitive behavioral game design framework (44). This framework involves the SCT, the Theory of Multiple Intelligences (MI), and gamification processes (44). MI tackles the multiple ways of achieving learning and experiences (music, mathematics, logic, humor, narratives) and highlights the game’s ability to use a multimodal approach, possibly combining sensoriality, emotions, and rational thinking (61). The author (44) summarized the challenge of game design in the question, “How do I express one or more social cognitive elements through the mechanism of one or more intelligences in a way that facilitates the enjoyment process?” thereby referring to the involvement of game theories in the design of SGs.

## Use Play, Fun, and Games Theories

It makes little sense to talk about gamification and then create something utterly boring. But how exactly does one create fun? The following three features are particularly helpful in developing a game process:

- The type of player: what is the target group more likely to engage in?
- Flow theory: full immersion and energized focus
- The player’s journey

The player’s journey is essential to gamification for structuring vision, content, and progression. Have you ever been on a website where you were overloaded with choice and yet had no idea how to find what you were looking for? Conversely, have you ever been on a website with little choice and without a search function, which did not help you to find what you were looking for? These examples both address the issues of purpose, autonomy, and mastery. Accordingly, if people have greater autonomy, but have no idea where they are heading—in other words, if they have little mastery—they find themselves in the first example. If they have a high level of mastery but little autonomy, they find themselves in the second example. The purpose in finding something needed on a web page is essential for a satisfying experience. Indeed, without purpose, there is no action (62).

When setting up and developing a solution, consideration of the gamer’s journey will help structure what you want users to find first. This will help you understand the basic features of the system that users might need to proceed to the next level, so that they do not feel over-challenged, but confident enough in their ability to take up the next challenge with mastery. These three features are closely related to one another for distinguishing what is boring from what is engaging: too much mastery with little autonomy is as boring as too much autonomy and a low level of mastery of what one is doing or supposed to do or achieve (6). According to Flow Theory and Motivation (63), activities that provide an optimal balance between challenge level and skill acquisition level create a motivational state of flow. The flow enhances immersion, creativity, and performance.

Building an attractive journey for the player with optimal flow further requires a sound knowledge of the audience’s perceived health-related needs, as well as of possible gamer types—e.g., an achiever is motivated by mastery, a socializer by social interactions, a philanthropist by purpose, and a free spirit by autonomy (64, 65).

## Be Mindful of the Social Link

According to Self-determination Theory (66), relatedness (e.g., the social link) is one of the keystones in behavior change processes. Many studies have shown the benefit of having a buddy, a community, or some kind of social support when one engages in a process, be it about improving wellness and fitness or dealing with a disease (39).

In games, it is easy to understand how these varied social interactions develop in a fun way. Amy Jo Kim—a game designer known for her career and work on games such as “The Sims”—has developed a social interaction chart (67) that shows the different ways in which people engage socially, some of which might seem more meaningful than others, depending on the type of player.

Social interactions may bring meaning into the system that you are implementing and may encourage bonding and a sense of belonging, which can be powerful for driving behavior change. In the game design process, social interactions can be divided into three categories corresponding to the following questions:

- Who are players sharing the experience with? A community? A chosen buddy? Their family?
- What feelings do you want players to derive from social links? Recognition? A sense of belonging to something bigger than themselves? Feedback? Naches (i.e., pride in the achievement of offspring or an apprentice, the joy that can be felt without doing anything directly)?
- Keeping the pursued goal in mind, what type of interactions are you looking for? As everyone likes to engage in interactions in different ways—although they might prefer one at a given time—they usually choose from several “interaction types.”

## It is Not Just about Game Mechanics and Game Dynamics

Gamification is about games and about making games appealing. Indeed, it is critical to keep in mind that the system has to be attractive enough for gamers to continue using it, even if points, badges and leader boards are removed (1). One should favor mechanics such as meaningful feedback and timely rewards that help gamers to improve their standing or to proceed to the next level more effectively (68). This will ensure that, besides appealing to users who favor extrinsic motivators (e.g., a Smart Box, tablets, etc.), your system will appeal to those who favor intrinsic motivators (e.g., learning) (69, 70) and may secure users’ engagement in the game for a longer time.

When designing games, one should also keep in mind that static things, although exciting at first, can easily lose their appeal. By way of illustration, the Fun Theory piano stair (71) is a cool way of making people choose regular stairs rather than the escalator; but would people feel the same way after 5 days? 10 days? Providing a game experience that is difficult enough for users to feel challenged (where they must make an effort to succeed), but easy enough for them to remain confident that they can succeed, may be the condition for keeping users engaged in the game. Conversely, providing a game experience where users repeat the same actions over and over may cause them to lose interest.

## Further Steps

Because further improvements in health-related games are needed, we encourage further studies to be done to explicitly describe the game design process, possibly by using the six tips described in the present paper. This may help to further refine game design taxonomy in the field. Assessing health-related outcomes that have already been achieved in a number of studies, as well as linking such outcomes to intra-game processes and gamers’ profiles, might also prove valuable.

## Author Contributions

We confirm that all authors contributed equally to the writing of this manuscript.

## Conflict of Interest Statement

AS is CEO of PlayBenefit and YK is one of the authors of the following games: “Michael’s Game” and “Pick-Klop.” The reviewer MP and handling editor declared their shared affiliation.
